# A Quality Improvement Assessment of the Delivery of Mental Health Services among WTC Responders Treated in the Community

**DOI:** 10.3390/ijerph16091536

**Published:** 2019-04-30

**Authors:** Mayer Bellehsen, Jacqueline Moline, Rehana Rasul, Kristin Bevilacqua, Samantha Schneider, Jason Kornrich, Rebecca M. Schwartz

**Affiliations:** 1 Department of Psychiatry, Unified Behavioral Health Center and World Trade Center Health Program, Northwell Health, 132 East Main Street, Bay Shore, NY 11706, USA; mbellehsen@northwell.edu; 2 Department of Occupational Medicine, Epidemiology and Prevention, Northwell Health, 175 Community Drive, Great Neck, NY 11021, USA; kbevilacqu@northwell.edu (K.B.); sschneide5@northwell.edu (S.S.); 3 Department of Biostatistics and Department of Occupational Medicine, Epidemiology and Prevention, Northwell Health, 175 Community Drive, Great Neck, NY 11021, USA; rrasul@northwell.edu; 4 World Trade Center Health Center, Northwell Health, 97-77 Queens Blvd, Rego Park, NY 11374, USA; jkornrich@northwell.edu; 5 Feinstein Institute for Medical Research, Department of Occupational Medicine, Epidemiology and Prevention and Northwell Health and Joint Center for Trauma, Disaster Health and Resilience at Mount Sinai, Stony Brook University, and Northwell Health, 175 Community Drive, Great Neck, NY 11021, USA; rschwartz3@northwell.edu

**Keywords:** disaster mental health, evidence-based treatment, mental health service utilization, quality improvement

## Abstract

The World Trade Center Health Program (WTCHP) provides mental health services through diverse service delivery mechanisms, however there are no current benchmarks to evaluate utilization or quality. This quality improvement (QI) initiative sought to examine the delivery and effectiveness of WTCHP mental health services for World Trade Center (WTC) responders who receive care through the Northwell Health Clinical Center of Excellence (CCE), and to characterize the delivery of evidence-based treatments (EBT) for mental health (MH) difficulties in this population. Methods include an analysis of QI data from the Northwell CCE, and annual WTCHP monitoring data for all responders certified for mental health treatment. Nearly 48.9% of enrolled responders with a WTC-certified diagnosis utilized treatment. The majority of treatment delivered was focused on WTC-related conditions. There was significant disagreement between provider-reported EBT use and independently-evaluated delivery of EBT (95.6% vs. 54.8%, *p* ≤ 0.001). EBT delivery was associated with a small decrease in Posttraumatic Stress Disorder (PTSD) symptoms over time. Providers engaged in the process of data collection, but there were challenges with adherence to outcome monitoring and goal setting. Data from this report can inform continued QI efforts in the WTCHP, as well as the implementation and evaluation of EBT.

## 1. Introduction

### 1.1. WTC Responders’ Mental Health

Following the September 11, 2001 attacks on the World Trade Center (WTC), an estimated 90,000 World Trade Center responders (WTC responders) provided emergency services at Ground Zero, where they were exposed to unprecedented traumatic events [1] while providing rescue, recovery, demolition, debris removal, and related support services in the aftermath of the attack. Symptoms of PTSD, depression, panic disorder, and anxiety have been observed in WTC responders over 10 years after the events of 9/11 [2,3,4,5,6,7,8,9,10,11,12,13,14]. 

The rate of PTSD among WTC responders continues to be significantly higher than in the general population, and is the most prevalent mental health (MH) diagnosis for responders and clean-up workers at Ground Zero [2]. One study found that 9.7% of WTC responders interviewed 11–13 years after 9/11 met the criteria for current PTSD [7], compared to the estimated lifetime PTSD prevalence in the general United States population of 6.4% [15]. Furthermore, a study of over 10,000 WTC responders found that nearly 7% experienced a delayed onset of symptoms up to 8 years after 9/11, and the risk of stable or worsening PTSD among first responders is supported in the literature [16].

Limited research has been conducted on treatment for PTSD in a first responder population [17]. Three studies have examined the efficacy of specific treatment modalities among WTC responders, including combined prolonged exposure and paroxetine [18], cognitive behavioral treatment [19], and virtual reality exposure therapy [20]. These studies have been limited in several important ways including the use of diverse exposure assessment tools limiting comparability and small sample sizes [21]. The generalizability of these studies to clinical settings is somewhat limited, in that these studies examine treatment outcomes within a clinical trial, and can speak to treatment efficacy. However, they do not necessarily reflect MH utilization and treatment outcomes among WTC responders obtaining routine treatment for PTSD, thereby hindering conclusions regarding clinical effectiveness. In addition, the studies were university-based, and not reflective of care provided in the community. To our knowledge, no studies have focused on the evaluation of treatments for other mental health difficulties (besides PTSD), specifically among WTC responders, including depression and anxiety [21]. Little is also known as to the extent to which WTC responders are obtaining recommended evidence-based treatments (EBTs) for their mental health diagnoses, including PTSD, or the impact of treatment upon their symptoms. 

### 1.2. Mental Health Service Provision for WTC Responders

The WTC Health Program (WTCHP) (previously known as the Medical Monitoring and Treatment Program) was established in 2002 to address the physical and mental health needs of WTC responders through annual medical monitoring, and then through the delivery of no-cost medical services [1]. WTC responders treated through the WTCHP (WTCHP responders) include traditional first responder professionals, such as police, paramedics and non-FDNY firefighters, as well as non-traditional responders like construction workers and vehicle maintenance workers. To date, an estimated 45,894 WTC responders have had at least one monitoring visit as part of the WTCHP. The structure of the program’s service delivery efforts rests on partnerships between hospital systems and community practitioners to meet the needs of the population. Clinical Centers of Excellence (CCE) are responsible for monitoring the well-being of participants, and then authorizing physical and mental health treatment in the community. Mental health services coordinated by CCEs are delivered through a provider network that is comprised of health system-attached clinics and private practice-based clinicians enrolled by the CCE. A recent report [22] indicated that in 2017, about 5% of continuously-enrolled WTCHP responders utilized MH services (excluding pharmacy), and 99% of these services were delivered in an outpatient setting. However, utilization rates varied among sub-groups of members and by each individual CCE, and little is known about the quality and effectiveness of the treatment provided. This gap warrants attention, given the high prevalence of PTSD, depression, and anxiety in this population.

Mental health programs and systems of care that have focused on improving the quality of services and effectiveness of treatment have broadly targeted two areas for intervention: Supporting evidence-based practice implementation (EBPI), and measurement-based quality improvement (MBQI) [23,24]. Treatment recommendations for PTSD include first-line recommendation on the delivery of trauma-focused EBTs (TF-EBTs), such as Prolonged Exposure therapy and Cognitive Processing Therapy [25,26]. Despite these recommendations, it is known that, in general, providers do not sufficiently use research to inform practice [27]. 

Prior to a heavy investment by the Veterans Administration (VA) to support implementation of EBTs for PTSD, one survey showed that as few as 10% of PTSD specialists and general MH providers utilized evidence-based treatments for PTSD [28]. Since the VA investment in implementation of TF-EBTs in 2006, progress has been made, yet penetration of these treatments is highly variable, and challenges remain [29,30]. 

### 1.3. Quality Improvement Initiative

The provision of no-cost MH services to a large cohort of responders and the longitudinal, standardized monitoring of a large cohort of WTC responders through the WTCHP presents a unique opportunity to better understand the delivery and effectiveness of mental health care services for this population. The current analysis aims to characterize the provision of mental health services to WTC responders within the Northwell Health Queens World Trade Center Health Program, also referred to as the Northwell Clinical Center of Excellence (CCE). Data for this analysis relies on a quality improvement project that was initiated in late 2017. It entails modification of treatment plan data collected in the course of administering the program to obtain information on the interventions being utilized, and to require greater movement towards measurement-based care. In addition, WTCHP annual monitoring and claims data can be used to verify the information and findings from the treatment plan data. The goal of the quality improvement project is to characterize the mental health services delivered to clients, to begin implementing an MBQI process, and to explore delivery of recommended EBT for PTSD and other WTC-certified mental health diagnoses to WTCHP responders being treated for mental health difficulties by community providers. Upon characterizing the population and understanding baseline data, program interventions can be designed to promote engagement in care, as well as enhanced services and outcomes for the population. 

## 2. Materials and Methods

### 2.1. Data Sources

This is a quality improvement initiative, focused on a subset of community mental health providers who provide psychotherapy to World Trade Center Health Program (WTCHP) responders (i.e., WTC responders to 9/11, excluding FDNY firefighters) served by the Northwell Center of Excellence (CCE). The Northwell CCE Treatment Plan Database was started in December 2017 to monitor mental health treatment delivery and outcomes, and consider targets for quality improvement. Community mental health providers for the Northwell CCE include psychiatrists, psychologists, and clinical social workers spread across the community, some of whom are attached to a Northwell Health ambulatory program. Due to logistical challenges, such as administrative burden, and the fact that the ambulatory programs have their own processes for QI, efforts were initiated with just psychotherapists located in private practices in the community. Within this group, 100% of providers were able to provide treatment plan data. The project was submitted to the Northwell Health IRB for evaluation as research, and deemed as non-human subjects research, due to its quality improvement (QI) focus. Providers had already been completing treatment plans regarding the mental health treatment of WTCHP responders, but these were not developed or analyzed in a systematic manner. Members of this team modified the plans to ensure that they include items to assess modality of treatment, descriptions of treatment, including whether or not the provider believed that the treatment was evidence based, goals for treatment, scores on standardized measures of symptoms, expected duration of treatment, and explanation of the connection between treatment and 9/11 exposure. Providers were engaged prior to the modifications of the treatment plans, and then again after the plans were modified, to inform them of changes to the treatment plans, and to answer questions. The Northwell CCE team was also available for questions as they arose. Follow-up treatment plans are completed every four months, and the data is inputted into the database by a Northwell CCE staff member. For the current analysis, data from baseline (Review 1) and the first four month follow-up time point (Review 2) were used. 

Data from annual monitoring visits collected for the WTCHP General Responder Cohort for all Northwell CCE responders certified for mental health treatment were also obtained and linked to the Treatment Plan Database, to provide annual survey and administrative claims data, which included demographics, mental health diagnoses, standardized scores of mental health symptoms, and mental healthcare utilization data. 

### 2.2. Treatment Plan Data

Data reported from the Northwell CCE community provider network included provider-reported used of evidence-based treatments (EBT) (yes/no), goals requested in a specific (or simple), measureable, achievable, relevant, and time-framed (SMART) format, treatment modality (i.e., individual weekly, family, group, medication management) type of therapy used, whether treatment was WTC-focused, and the expected length of further treatment. Mental health symptom scores were also reported.

*Primary variables of interest:* To evaluate whether recommended EBT was delivered to the WTCHP responder, treatment plans were reviewed by two independent psychologists with backgrounds in EBT for mental health (MH) disorders, with particular expertise in working with responders. A standardized decision process was formulated to make this decision. This included alignment between diagnosis and indicated EBT, that the provider was utilizing as per the American Psychological Association (APA), Veterans Administration/Department Of Defense (VA/DOD), and National Institute for Health and Care Excellence treatment recommendations, where available [31,32,33]. Information from other sections of the treatment plan was also utilized. For example, reference to exposure-based goals (for Posttraumatic Stress Disorder (PTSD)) was indicative of some EBT use. Therapy was categorized by reviewers as: Cognitive Behavioral Therapy (CBT), Trauma-Focused Evidence-Based Therapies (e.g., Prolonged Exposure, Cognitive Processing Therapy etc.), non-trauma focused evidence-based therapies for PTSD (e.g., Interpersonal Psychotherapy, Stress Inoculation Therapy, etc.), eclectic/supportive therapy, mindfulness/relaxation therapy, psychodynamic therapy, and non-recommended therapies for PTSD (e.g., “Emotional Freedom Technique”). The determinations for which therapies are considered recommended for PTSD, were those based on the treatment guidelines noted above. Therapies which did not meet any criteria for a recommendation based on these guidelines may still have an evidence base, but it was categorized as non-recommended. The variable (i.e., independent reviewer assessment of recommended EBT delivery) was labeled *EBT Delivered* and categorized as: Yes, No, Elements of EBT-Delivered, and Cannot Interpret from Clinician’s Report. For analysis, EBT-Delivered was dichotomized as Yes (Yes or Elements of EBT-Delivered), or No. Cases where EBT-Delivered could not be interpreted from the clinician’s report (*n* = 4), were excluded for analysis for this variable.

Reviewers also independently evaluated provider-reported therapy, use of SMART goals, and whether or not treatment had a WTC focus. Providers were expected to list goals in SMART format, which is frequently used as a frame for goal setting across disciplines. This format facilitates the reporting of measurable progress for WTCHP responders, even in the absence of EBT and standardized symptom measurement. Providers were also asked to elaborate on whether or not their treatment had a WTC focus, which was then reviewed.

Lastly, providers were asked to include symptom severity scores by utilizing self-report instruments with clients, summing the items to calculate symptom scores, and then including the final score in their treatment plans. For PTSD symptom severity, providers used the Posttraumatic Stress Disorder Checklist-5 (PCL-5)—a 20 item measure of PTSD (range = 0–80) [34]. For depression symptom severity, providers used the Patient Health Questionnaire-9 (PHQ-9)—a nine item measure of depressive symptoms (range = 0–27) [35]. For anxiety symptom severity, providers utilized the General Anxiety Disorder-7 Item (GAD-7)—a seven item measure of anxiety symptoms [36].

### 2.3. WTCHP Annual Medical-Monitoring Data

Mental healthcare utilization was extracted from administrative claims out of the WTC annual monitoring data for all Northwell CCE responders certified for MH (*N* = 477). Utilization during 2018 was defined as having at least one claim for psychotherapeutic services, diagnostic evaluation, or medication management. Psychotherapy treatment modality (individual, family), length of treatment session (20–30 min, 45–60 min), and number of medication management appointments, were also tabulated. The number of sessions per WTCHP responder within each year was also determined. 

The WTCHP Annual Medical-Monitoring data also provided demographics including age, sex (male/female), race, ethnicity, and education (<high school (HS), ≥HS). ICD-9 and ICD-10 codes were extracted to define WTC-related certified mental health diagnoses of PTSD, anxiety and depression. PTSD, anxiety and depression symptom scores were measured annually, using the PCL-S (specific to the WTC terrorist attacks on 9/11), PHQ-9, and GAD-7 scales, respectively. For each instrument separately, items were summed to create symptom severity scores for PTSD (range = 17–85) [37], depression (range = 0–27) [35], and anxiety (range = 0–21) [36]. In our sample, the reliability of these instruments was high (Cronbach’s alpha: PCL-S = 0.949, GAD-7 = 0.914, PHQ-9 = 0.921).

### 2.4. Statistical Analysis

From the WTCHP Annual medical-monitoring data, mental healthcare utilization rates and number of sessions per WTCHP responder were described for 2018 overall, and for the responders treated by the Northwell CCE community provider network. In the Treatment Plan Database, WTCHP responder characteristics at first review were compared by EBT-Delivered, using the Mann-Whitney Rank Sum test for continuous variables, and Fisher’s exact test for categorical variables. McNemar’s test and the Kappa statistic were used to evaluate any agreement between provider-reported EBT and EBT-Delivered.

For WTCHP responders where MH symptom scores at both review 1 and 2 were available, the change in mental health symptom scores between the reviews were described using mean differences overall, and were stratified by EBT-Delivered. To assess the sensitivity of the Treatment Plan Database results, the change between MH symptom scores between the last two annual monitoring visits from the WTC-data monitoring data, was also analyzed. For this analysis, WTCHP responders from the Treatment Plan Database were linked to their annual monitoring visit data, and included if they had two annual visits from 2016 to 2018 (*N* = 86). Analyses were conducted using SAS software, version 9.4 (SAS Institute, Cary, NC, USA). 

## 3. Results

Of the 477 WTCHP responders certified for MH, 48.9% utilized mental healthcare in 2018 (Table 1). WTCHP responders utilized: Diagnostic evaluation (14.9%), psychotherapeutic services (41.3%), and/or medication management (8.2%). Of WTCHP responders who utilized psychotherapeutic services (*n* = 197), all had individual sessions, and 92.4% of them lasted between 45–60 min. Only 3% also had a family session. About one third of WTCHP responders (35.5%) also had diagnostic evaluation or medication management. WTCHP responders had a median number of 17 (IQR = 5–34) individual therapy sessions in 2018. As expected, utilization rates were high among WTCHP responders captured through the Treatment Plan Database.

From the Northwell Treatment Plan Database, data were collected from 16 providers on 129 WTCHP responders at the first review, and of those, 103 had a second review. During the four month review period, providers conducted a median of 11 sessions (IQR = 2–37, range = 1–64), and saw a median of 5.6 (range = 1–29) WTCHP responders. WTCHP responders were 55.3 (SD = 9.6) years old on average, 32.8% female, and 40.2% white (Table 2). Most were diagnosed with PTSD (74.4%) and some had anxiety (24.8%) and depression (34.1%) diagnoses. The majority underwent weekly individual therapy (72.1%), and the estimated length of further treatment was 12 months for 61.7% of WTCHP responders. Two WTCHP responders were indicated to have group therapy as well. 

Providers reported that nearly 94% of services delivered were evidence-based treatments with the majority of providers reporting delivering CBT (74.4%), and mindfulness or relaxation therapy (19.4%). Notably, despite reporting delivery of EBTs, providers also reported delivering eclectic/supportive therapy (which are not categorized as EBTs) 23.3% of the time. Based on the independent QI review, treatment was determined to be largely WTC-focused (85.3%). However, some of the provider responses were characterized as “unclear” (14%).

Almost all providers in the community provider network (96.0) reported using EBT. For those providers that indicated the use of EBT, treatment plans were reviewed by independent Northwell CCE reviewers to determine EBT-Delivered status. Sixteen WTCHP responders (12.4%) were assessed to have likely received EBT, and 52 (40.3%) were assessed to have likely received elements of EBT at baseline. There was also a significant disagreement between provider-reported EBT use and an independently-evaluated delivery of EBT (95.6% vs. 54.8%, McNemar’s test *p* < 0.001, Kappa coefficient = 0.097 (95% CI = 0.015–0.179)). Demographics and WTC-certified diagnoses were similar across WTCHP responders, with EBT-Delivered compared to not (Table 2). Use of SMART goals (70.6% vs. 24.6%) and number of SMART goals ≥3 (89.71% vs. 59.7%), were more frequent with patients who had EBT delivery. Additionally, when EBT was not delivered, WTCHP responders had higher levels of missing documentation of goal progress (35.1% vs. 14.7%), and a longer length of further treatment (80.7% vs. 43.9%).

Among all WTCHP responders where MH symptom scores at both review 1 and 2 were available, PTSD anxiety, and depression symptom scores improved by 1.81, 0.46, and 0.03 points, respectively. When compared by EBT Delivered, similar changes were noted. PTSD symptom scores decreased 2.29 points in the four month period for those with EBT-Delivered. There was also a weaker mean decrease of 1.42 points among the group without EBP delivery. WTCHP responders with and without EBT-Delivered also had small decreases from review 1 to review 2 in mean anxiety and depression symptom scores (Table 3). The analysis of the WTC annual monitoring data also indicated small symptom changes over time by EBT provision (Table 3). 

## 4. Discussion

This project has yielded numerous findings and considerations for future efforts. Consistent with a recent HRA report on service utilization, our team found that among WTCHP responders with a WTC-certified mental health diagnosis, nearly 49% utilized mental health care [22]. That report found the Northwell CCE had service utilization of 55%, but their analysis included the removal of responders that may have unenrolled in the program, or were deceased. This information was not available to us for this project, and it is likely that if this information were accounted for, the overall cohort would shrink, and the utilization rate would further rise. This level of service utilization meets the national average [22] for access indicated in the HRA report, and speaks to the value of using a community provider network to ensure access for WTCHP responders. 

It was also meaningful that most providers (85%) were providing treatment that was deemed as targeted towards the WTC-attributed diagnosis. Consistent with the literature, the most prominent MH diagnosis for the overall cohort at the Northwell CCE was PTSD. Most providers indicated that they were working on goals that were related to the sequelae of 9/11, such as PTSD and its associated impact on interpersonal functioning. This is a difficult variable to assess, as treatment is not being directly observed, but these findings are promising for adherence to WTCHP objectives. 

This QI project was also notable for the high rate of participation by providers in the process. The providers were familiar with completing some information for treatment plans as part of past program expectations, but this project included a more robust overhaul of the treatment plan process. There were challenges with framing goals in a SMART format, and with ensuring the collection of all outcome data, but most providers completed numerous data elements for the treatment plans, despite the extra reporting requirements. For example, almost no providers submitted treatment plans without goals, and the majority of providers documented progress on at least one goal. Even among providers who had missing outcome measures, many of these providers completed some data points. Importantly, the providers whose treatment plans were evaluated, were also engaged in the development of the plans. Quality improvement is seen as a bottom up process, and stakeholder engagement is therefore critical to implementing systems change, which likely contributed to this high participation [24]. 

The finding that there was an association among providers between delivering EBT services and a greater use of SMART goals was interesting. Without further exploration into this association, it is difficult to say why this occurred, but it may reflect some underlying shared attribute among those providers towards utilization of measurable objectives and provision of EBT services. Frequent utilization of measurement tools is consistent with expectations of EBTs, and may help explain this association. Informal conversations with providers who had difficulty around both the SMART goal format and reporting on outcome measures, indicated numerous challenges, such as confusion about what SMART formats entailed, and challenges obtaining measures from WTCHP responders. Future efforts of this QI project should include further education of providers on how to meet these types of objectives and engage with providers on problem solving challenges to obtain outcome measurements. 

Interestingly, nearly all providers in this project claimed to be providing EBT to the majority of their clients. This is a higher number than expected, and was unforeseen for numerous reasons. As noted earlier, even when providers are trained in EBTs, and are provided institutional support to execute these treatments, penetration is challenging [29]. Furthermore, it has been often reported that there is a gap between clinical research and practice [27]. Additionally, while providers endorsed utilizing an EBT, a significant number of them endorsed utilizing eclectic/supportive therapies, and many indicated utilizing therapies for PTSD that are not recommended as EBTs for PTSD. It is therefore not surprising that upon independent review by Northwell CCE psychologists, there was significant disagreement with the provider report. Only about 12% of WTCHP responders were deemed to have likely been in receipt of a recommended EBT, and an additional 40% were considered to have likely received some elements of an EBT, such as cognitive restructuring or exposure therapy. Notably, even these numbers may be an over-representation of true EBT delivery, as treatment was not observed directly, and no treatment fidelity checks were in place. 

Additionally, many of the clients were deemed by providers to require an additional 12 months of treatment, and the median number of sessions per WTCHP annual monitoring data for 2018 was 27 sessions. This would be inconsistent with expectations of many EBTs that typically range from 8 to 20 sessions. Accurate assessment of EBT delivery is a challenging endeavor that will continue to require attention if this is an area of priority for the WTCHP. 

When recommended EBT or components of EBT were deemed to have been delivered, the WTCHP responders demonstrated small improvements in measures of PTSD symptoms. This was notable for PCL-5 scores that were attained using the Treatment Plan Database, however, these changes were below the 10 point change that is considered a clinically significant change [38]. Additionally, no significant changes in PTSD symptoms were observed with the data collected from the annual monitoring exam. While the time between measurements on the annual monitoring exam is about one year, as compared to the four months in the Treatment Plan Database, the overall negligible movement in the measurement of symptoms warrants further exploration. It may be that even in cases of clients deemed to be receiving recommended EBTs, they are not receiving them with great fidelity, which speaks to program effectiveness, as opposed to efficacy. If this is the case, greater investment on the part of WTCHP in supporting the dissemination of EBTs for this cohort may lead to an improved delivery of these treatments.

The findings from this report suggest that future QI projects within the Northwell CCE should explore mechanisms for increasing engagement in outcome monitoring and support of EBT interventions. Additionally, future research among the Northwell CCE clients, and in the larger WTCHP mental health cohort, is needed to explore patterns of change in outcomes for those engaged in treatment. While those in treatment may not be demonstrating substantial improvements in symptoms on self-report measures, those who are being seen for an extended duration may be obtaining maintenance treatment that could be preventing any exacerbation of symptoms. It may also be the case that treatment is not being delivered as effectively as desired, or that there are unique challenges to understand with this responder cohort, which explain the lack of demonstrable progress on these measures. For instance, this population may have a high degree of medical co-morbidity, as well as exposure to multiple and severe traumas, that may be hindering the treatment progress [7,13]. 

Several limitations should be noted. Firstly, this is a quality improvement project that included an analysis of our institution’s administrative data, and should be interpreted as such. We did not formally adjust significance values for multiple comparisons. Secondly, not all providers overseen by the Northwell CCE were included at this step of the QI process, and may not be accessible without further administrative mandates, given that the providers who did not participate were also part of other systems of care with a high level of administrative burden. This made it difficult to obtain the necessary information without adding to the administrative burden of those providers. Accordingly, the data obtained could reflect a potential response bias. Thirdly, even among those who did complete treatment plan data, the information provided was the report of the provider, and not from direct observation. Specifically, outcome data were self-report measures that providers collected, scored, and then reported on, as opposed to data to which the team had direct access. Furthermore, EBT delivery was captured through an analysis of treatment plans, as opposed to fidelity checks of treatment which would have been prohibitive. These issues were compensated for by evaluating data from the annual monitoring report, where feasible. Finally, a high level of missing data was present for mental health outcomes, and we were not able to model changes in outcomes. Instead, unadjusted analyses were used to compare differences in symptom scores across time, and whether those changes were different by use of EBT Delivered. This hampered our ability to adjust for potential confounding variables as well. Therefore, the results from the analysis represent a signal to be explored further to determine if it reflects the larger treatment plan population, or the WTC responder population more broadly. 

Many of the limitations listed above are to be expected in a QI project that does not allow for rigorous controls of treatment setting and delivery. Instead, this project reflects a real-world delivery setting for services, and offers meaningful first steps towards assuring quality of treatment in a WTCHP CCE, that could have implications for other CCEs and the larger MH treatment population. Untreated PTSD and other mental health conditions can have a debilitating impact on functioning, and come at a significant cost to society [39]. It is therefore important that the CCEs consider opportunities for improving services among their providers. These services are often delivered in very different contexts, such as in ambulatory programs of hospital systems, and in private practices of community providers. Systems of care and individuals can vary widely in the quality and consistency of the services delivered. This poses a challenge in assuring the quality of services for enrollees in the WTCHP both across and within CCEs.

The Substance Abuse and Mental Health Services Administration (SAMHSA) has directed significant attention towards supporting the adoption of evidence-based practices and collecting and utilizing data to inform policy and practice [40]. Progress has also been made towards a broad standardization of outcome measures, such as those undertaken in collaboration with the National Quality Forum [41] and the National Committee for Quality Assurance [42]. The WTC programs, given their unique funding structure, allow for greater flexibility and oversight to develop QI initiatives and encourage provision of EBTs by community providers, who provide the care, with the assurance that they will be paid for their clinical service.

Should the WTCHP move towards more standardized metrics for MH, and/or support for any implementation of evidence-based therapies, QI processes are an ideal mechanism for measurement and intervention [23,24]. Unfortunately, it is unclear as to what would constitute ideal targets for an improvement of program performance. As noted, mental health utilization in the Northwell CCE meets the national average target, but it is not clear as to what would be the ideal performance measures for outcome monitoring or EBT delivery. In addition, demands and priorities should be sensitive to the contexts in which they are implemented. It is important to bear in mind that even robust implementation programs have had significant challenges. Furthermore, EBT implementation and quality assurance processes have greater chances of success when the stakeholders’ voices and the contexts of implementation are factored into the process of change [43,44]. Due to the various demands imposed on providers, and the diverse contexts within which the WTCHP operates, modifications should be selective in targeting particular outcomes that will have the greatest value to the WTC MH cohort. Given the high proportion of responders with PTSD, this will likely require consideration of interventions that enhance services and outcomes for that population. 

## 5. Conclusions

This project has leveraged the usage of quality improvement mechanisms to characterize service delivery, evaluate both EBT provision and the implementation of outcome monitoring in a community provider network for the WTCHP. The initial findings include: Robust access to psychotherapeutic services, adherence to program objectives of treating WTC-related conditions, and an engagement of providers in the process. However, the findings also point towards areas for improvement in both EBT delivery and measurement based care, with implications for the well-being of members served. Priorities for program performance need to be set by the WTCHP, but information gleaned from this project in the Northwell CCE provide some areas for consideration. Providers could be engaged in more outcome-based measurement, and could be given more support for EBT training and delivery. Close coordination of these changes with relevant stakeholders (i.e., CCEs, providers, and clients) could ensure effective implementation of any desired objectives.

## Figures and Tables

**Table 1:** Mental healthcare utilization by service type from January 2018–December 2018.

Type	Northwell Health CCE Responders (*N* = 477)			Community Provider Network (*N* = 124 ^a^)		
	*n*	%	No. Sessions/WTCHPR Med (IQR)	*n*	% ^b^	No. Sessions/WTCHPR Med (IQR)
Overall MH Utilization	233	48.9	13 (3–30)	118	95.2	27 (15–43)
*Diagnostic Evaluation*	71	14.9	1 (1–2)	7	5.7	2 (1–2)
*Medication Management*	39	8.2	4 (2–8)	19	15.3	6 (2–8)
*Psychotherapeutic Services*	197	41.3	17 (5–34)	117	94.4	27 (16–40)
Family	6	1.3	1 (1–2)	6	4.8	1 (1–2)
Individual	197	41.3	15 (5–34)	117	94.4	26 (13–40)
20–30 min TX	37	7.8	2 (1–4)	15	12.1	3 (1–4)
45–60 min TX	182	38.2	18.5 (6–35)	114	91.9	27 (15–40)

MH = mental health. TX = treatment. No. = number. WTCHPR = World Trade Center Health Program Responder. Med = median. IQR = Interquartile range. ^a^ Of the 129 WTCHP responders in the Treatment Database, those who were transferred out of the Northwell CCE were not included in the WTCHP Annual Monitoring data (*n* = 5). ^b^ Mental healthcare utilization was not 100% for WTCHP responders in the Treatment Database. This may be due to the lag time in processing claims in the annual monitoring data.

**Table 2:** Characteristics (%) compared by reviewer-assessed EBT delivery.

Variable	Category	Total (*N* = 129)		EBT = No (*N* = 57) ^a^		EBT = Yes (*N* = 68) ^a^		*p* Value
		*n*	%	*n*	%	*n*	%	
**Demographics**								
Age, mean (SD) ^b^		55.3 (9.6)		55.1 (11.6)		55.1 (7.8)		0.984
Gender	Male	82	67.2	38	71.7	40	61.5	0.329
	Female	40	32.8	15	28.3	25	38.5	
Race ^c^	White or Caucasian	49	40.2	22	41.5	26	40.0	0.244
	Black or African American	20	16.4	10	18.9	8	12.3	
	Other/Unknown	53	43.5	21	39.7	31	47.7	
Ethnicity ^c^	Non-Hispanic	48	53.3	24	64.9	22	44.0	0.082
	Hispanic	42	46.7	13	35.1	28	56.0	
Education ^c^	<HS	17	15.9	5	11.4	12	20.3	0.288
	≥HS	90	84.1	39	88.6	47	79.7	
Language ^c^	English	95	78.5	47	88.7	45	70.3	**0.023**
	Spanish	26	21.5	6	11.3	19	29.7	
**Certified MH Conditions**								
Anxiety	No	97	75.2	47	82.5	47	69.1	0.099
	Yes	32	24.8	10	17.5	21	30.9	
Depression	No	85	65.9	35	61.4	49	72.1	0.252
	Yes	44	34.1	22	38.6	19	27.9	
PTSD	No	33	25.6	12	21.1	20	29.4	0.311
	Yes	96	74.4	45	79.0	48	70.6	
**Treatment Related**								
World Trade Center (WTC) Focus ^c^	Yes	110	85.3	39	68.4	67	98.5	**<0.001**
	No	1	0.8	1	1.8	0	0.0	
	Unclear	18	14.0	17	29.8	1	1.5	
SMART Goals present ^c^	SMART Goals Present	25	19.4	3	5.3	22	32.4	**<0.001**
	Some SMART Goals Present	37	28.7	11	19.3	26	38.2	
	Goals, Not SMART	42	32.6	32	56.1	9	13.2	
	Goals, partially SMART	22	17.1	9	15.8	11	16.2	
	No Goals	3	2.3	2	3.5	0	0.0	
Number of SMART Goals	<3	33	25.6	23	40.4	7	10.3	**<0.001**
	≥3	96	74.4	34	59.7	61	89.7	
Length of further treatment (months) ^c^	≤8	48	37.8	11	19.3	37	56.1	**<0.001**
	≥12	79	62.2	46	80.7	29	43.9	
Documented Progress on ≥ 1 goal ^c^	Yes	66	51.6	29	50.9	35	51.5	**0.001**
	No	31	24.2	20	35.1	10	14.7	
	Can’t Interpret from clinician’s report	19	14.8	8	14.0	11	16.2	
	Initial Goal	12	9.4	0	0.0	12	17.7	
Treatment type ^c^	Weekly individual therapy	93	72.1	43	75.4	46	67.7	0.428
	<Weekly individual therapy	36	27.9	14	24.6	22	32.4	

EBT = reviewer assessed evidence-based treatment. SD = standard deviation. MH = mental health. HS = high school. PTSD = Post-traumatic stress disorder. SA = substance abuse. TX = treatment. Med = median. IQR = Interquartile range. ^a^ Four WTCHP responders are missing an EBP-Delivered classification, because reviewers could not determine it from the clinician’s report. ^b^ *p* Value for continuous variables based on the Mann Whitney Rank Sum test. ^c^ *p* Value for categorical variables based on Fisher’s exact test. Bold: *p* < 0.05.

**Table 3:** Mental health symptom scores at review 1 and review 2 compared by study variables.

	MH Symptom Score	EBT	*N*	Period 1	Period 2	Mean Change
				Mean (SD)	Mean (SD)	
Using TX Plan Data with Four Month Follow Up	PTSD symptom score (using PCL-5)	No	24	33.04 (17.21)	31.63 (17.12)	−1.42
		Yes	28	38.61 (16.09)	36.32 (15.76)	−2.29
	Anxiety symptom score	No	27	9.56 (5.06)	9.44 (5.44)	−0.11
		Yes	25	12.44 (5.51)	11.56 (5.42)	−0.88
	Depression symptom score	No	27	9.81 (5.21)	9.74 (5.1)	−0.07
		Yes	24	10.54 (5.45)	10.38 (5.25)	−0.17
Using Monitoring Data with One Year Follow Up	PTSD symptom score (using PCL-S)	No	28	43.52 (15.25)	45.16 (16.01)	1.64
		Yes	33	53.06 (13.17)	55.41 (13.74)	2.35
	Anxiety symptom score	No	17	8.53 (4.05)	8.18 (4.79)	−0.35
		Yes	24	9.71 (4.29)	10 (3.6)	0.29
	Depression symptom score	No	27	9.08 (5.32)	8.34 (5.23)	−0.75
		Yes	30	10.26 (5.84)	10.24 (6.06)	−0.02

MH = mental health. EBT = reviewer-assessed evidence based treatment. Med = Median. IQR = Interquartile range. PTSD = Posttraumatic stress disorder. TX = treatment. PCL-5 = Posttraumatic Stress Disorder Checklist-5.
